# The adipokine fatty-acid binding protein 4 and cardiac remodeling

**DOI:** 10.1186/s12933-020-01080-x

**Published:** 2020-07-29

**Authors:** Beatrice von Jeinsen, Lisa Ritzen, Julia Vietheer, Claudia Unbehaun, Maren Weferling, Christoph Liebetrau, Christian W. Hamm, Andreas Rolf, Till Keller

**Affiliations:** 1 Department of Cardiology, Kerckhoff Heart Center, Benekestrasse 2-8, 61231 Bad Nauheim, Germany; 2 Department of Internal Medicine I, Cardiology, Justus-Liebig-University Giessen, Klinikstrasse 33, 35392 Giessen, Germany; 3 German Centre for Cardiovascular Research (DZHK), Center Rhein-Main, Berlin, Germany

**Keywords:** Fatty-acid binding protein 4 (FABP4), Obesity, Adipokines, Left-ventricular hypertrophy, Cardiac magnet resonance imaging, CMR

## Abstract

**Background:**

Previous publications about the association between fatty-acid binding protein 4 (FABP4) and cardiac remodeling have reported different, both beneficial and harmful, associations. Aim of the present investigation was to evaluate the association of FABP4 with parameters of myocardial remodeling defined by cardiac magnetic resonance imaging (CMR).

**Methods:**

We investigated plasma FABP4 levels in 331 patients (71% men, mean age 63±13 years) with preserved left ventricular ejection fraction (LVEF ≥ 55%) who underwent a CMR examination. We used linear cox regression to investigate associations between FABP4 and left ventricular end-diastolic diameter (LVEDD), right ventricular end-diastolic diameter (RVEDD), relative wall thickness (RWT), left ventricular mass index (LVMI), and LVEF (unadjusted and adjusted for age, sex, body mass index, cardiac biomarkers, and comorbidities).

**Results:**

FABP4 levels were associated with lower LVMI and higher NT-proBNP levels in an adjusted model. The inverse association between FABP4 and LVMI was more pronounced in lower FABP4 levels, whereas the positive association between FABP4 and NT-proBNP was more pronounced in relatively high NT-proBNP levels.

**Conclusions:**

Possible beneficial and harmful associations between FABP4 and left ventricular size have been reported. Our results suggest a beneficial association with LVMI (more pronounced in lower FABP4 levels) but a harmful association with NT-proBNP (more pronounced in higher FABP4 levels). Therefore, our results might indicate a potential dose-dependent association of FABP4, but this observation needs further investigation in larger study samples.

## Background

Fatty-acid binding proteins (FABP) are intracellular lipid chaperones which bind hydrophobic molecules and assists their transport through membranes [1]. A member of this family is FABP4. FABP4 is mainly expressed in adipose tissue and macrophages and is closely linked with inflammation [2]. Unfavorable associations between FABP 4 and insulin resistance [3], diabetes mellitus [4], gestational diabetes [5], and the metabolic syndrome [6], have been reported and FABP4 is further more associated with atherosclerosis and cardiovascular diseases [1, 7]. Recent studies have reported, that FABP4 mRNA expression is higher in epicardial compared to subcutaneous fat, that FABP4 mRNA expression is associated with the extend of atherosclerosis in patients with metabolic syndrome undergoing coronary bypass surgery [8], that peripheral FABP4 levels are correlated to the extent of left atrial adipose tissue volume in patients undergoing ablation of atrial fibrillation, that FABP4 is a good marker to predict recurrence of atrial fibrillation after ablation [9], and that sodium glucose cotransporter 2 inhibitors decrease proinflammatory gene expression including FABP4 in perivascular adipose tissue [10].

The association between FABP4 and cardiac size and myocardial function however is still a matter of debate. On the one hand there is overwhelming evidence that FABP4 has adverse effects. FABP4 suppresses contraction of cardiomyocytes in a rodent model [11] and FABP4 might increase intracellular lipid accumulation in patients with type 2 diabetes leading to myocardial dysfunction [12]. FABP4 levels were higher in heart failure patients compared to patients without heart failure and correlated with heart failure severity and NT-proBNP levels [13, 14], and FABP4 is associated with incidence of heart failure [15]. FABP4 was higher in morbidly obese with diastolic dysfunction compared to morbidly obese without diastolic dysfunction [16], FABP4 was associated with diastolic dysfunction in a small population-based cohort [17], and FABP4 was associated with left ventricular mass and function in patients with sleep apnea [18], in obese women [19], and in patients with coronary artery disease [20].

On the other hand, while the above mentioned studies were carried out in smaller cohorts and in certain patient populations only, one recent publication in a large population-based cohort reported a potential favorable association between FABP4 and left ventricular mass [21].

Due to the well-known adverse effects of FABP4, FABP4-inhibition or FABP4-receptor-blocking has been discussed as possible therapeutic target in humans [22]. It is therefore crucial to understand if FAPB4 might also have potential beneficial effects on the heart. Aim of the present study was to investigate and to gain further insights into the associations between FABP4 levels and cardiac size and function, measured by state-of-the-art cardiac magnetic resonance imaging (CMR).

## Methods

### Study sample

Participants have been recruited from the BioCVI (cardiovascular imaging) cohort. The BioCVI study is an ongoing cross-sectional cohort study that started 2016 based in Bad Nauheim (Germany) as part of the Kerckhoff Biomarker Registry (BioReg). This study included patients with clinical indication for CMR and who were older than 18 years. All patients gave written informed consent and the study was approved by the local ethics committee.

**Fig. 1:**
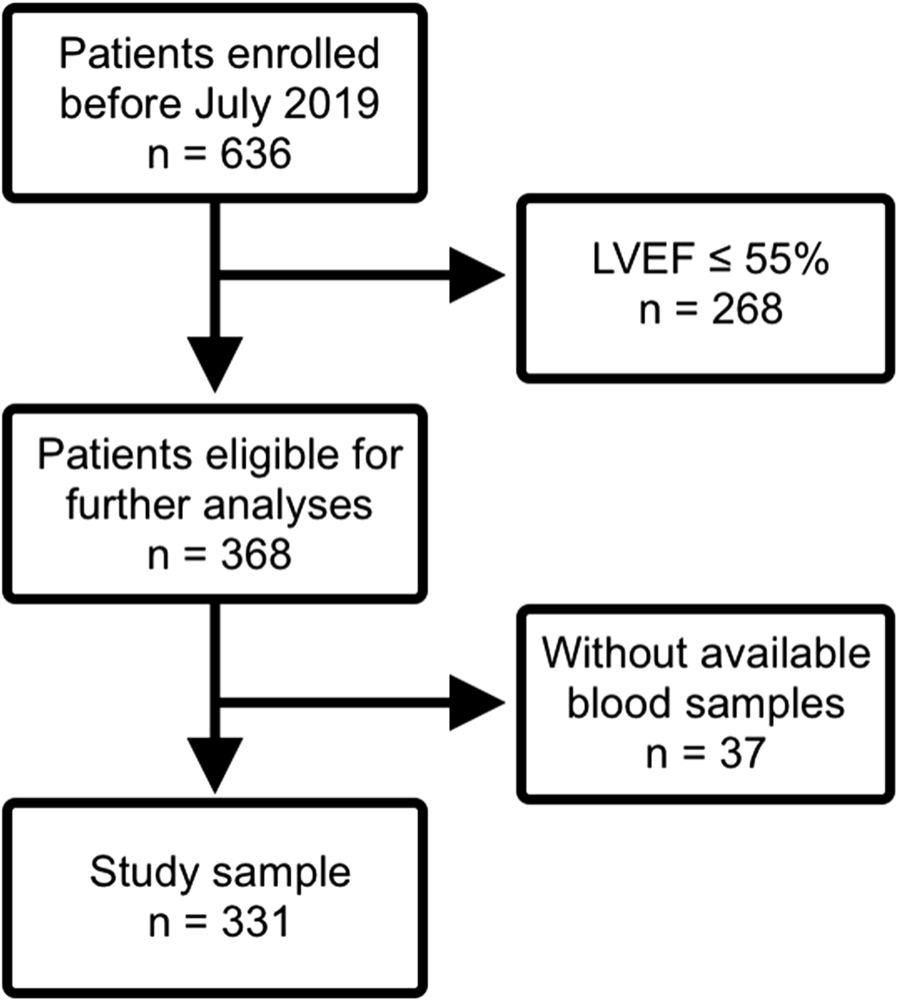
In- and exclusion criteria. Participants of the study with in- and exclusion criteria

All patients enrolled between August 2016 to July 2019 (n = 636) with suspected coronary artery disease or with suspected progress of an established coronary artery disease who therefore underwent CMR stress testing were eligible for further investigation. For the present analyses we excluded patients with prevalent heart failure (left ventricular ejection fraction [LVEF] < 55% (n = 268)). Further, patients without available blood samples for FABP4 measurements were excluded (n = 37) which lead to a final study sample of n = 331 patients (Fig. 1).

### Measurements of covariates

The baseline characteristics including the following were obtained at the point of study inclusion: sex, age, heart rate, weight, and height. Body mass index (BMI) was calculated based on height and weight (weight [kg]/height [m]^2^). Arterial hypertension was defined as systolic blood pressures ≥ 140 mm Hg and diastolic ≥ 90 mm Hg or the intake of antihypertensives. Diabetes mellitus was defined as fasting concentration of blood sugar ≥ 126 mg/dl, non-fasting blood sugar ≥ 200 mg/dl or the intake of antidiabetics. Former smoking is defined as non-smoking for more than 6 months. Coronary artery disease is defined as atherosclerotic changes of the coronary vessels. Atrial fibrillation was defined if present on the initial electrocardiogram.

### Blood sampling and laboratory measures

Venous blood samples were taken at the point of study inclusion. Routine laboratory parameters such as serum creatinine, C-reactive Protein (CRP), n-terminal pro B-type natriuretic peptide (NT-proBNP), and cardiac high sensitivity troponin T (hs-cTnT) levels were measured directly via the central laboratory. Additionally, venous blood samples were obtained prior the CMR examination, frozen and stored at − 80 degree Celsius for further measurements.

Plasma blood concentrations of FABP4 were measured by means of (sandwich) ELISA (enzyme-linked immunosorbent assay) (BioVendor). Interassay and intraassay variation of coefficients can be estimated at five percent.

### Measurement of CMR variables

The following CMR determined parameters were used for the present analyses: left ventricular end-diastolic diameter (LVEDD, mm), right ventricular end-diastolic diameter (RVEDD, mm), relative wall thickness (RWT, calculated by 2*posterior wall thickness/LVEDD), LVEF (%), and LVMI (g/m2), presence of late gadolinium enhancement, and presence of perfusion deficit.

### Statistical analyses

Baseline characteristics with normal distribution were characterized by arithmetic mean and standard deviation, whereas skewed variables were described by median and interquartile range. FABP4 levels were natural-logarithmically transformed to normalize its skewed distribution. Baseline characteristics were generated separately for men and women. They were furthermore generated stratified by FABP4 levels below or above the median and below and above the sex-specific median. The respective percentages, means and medians were compared using two-sided t-test or Wilcoxon rank test as appropriate. Spearman rank correlation analysis was carried out to investigate the correlations between FABP4 and covariates in the overall cohort, separately in men and women, as well as stratified by FABP4 levels above and below the median.

Multivariable linear regression was performed to study the association between FABP4 (independent variable) levels and CMR measures (dependent variables, RWT, LVEDD, RVEDD, LVEF, LVMI) individually. As we investigated five CMR measures, a Bonferroni corrected p-value of 0.01 was used to account for multiple statistical testing. Multivariable regression was performed unadjusted and adjusted for age, sex, body mass index, heart rate, estimated glomerular filtration rate (eGFR), NT-proBNP levels, and diagnosis of diabetes mellitus, arterial hypertension, or coronary artery disease. In secondary analyses the association between FABP4 and the cardiac biomarkers NT-proBNP and hs-cTnT was investigated. All analyses were carried out in the overall cohort, separately in men and women, as well as stratified by FABP4 levels above and below the median.

All analyses were performed using the R 3.4.1 software package (R Foundation for Statistical Computing, Vienna, Austria).

## Results

### Baseline characteristic

The baseline characteristics are presented in Table 1. Out of 331 patients, 95 patients (29%) were female, mean age was 63 years. Most of the patients had a history of arterial hypertension (75%). Male patients were more likely than female patients to suffer from coronary artery disease (48% vs. 28%, p = 0.002) and had a higher rate of late gadolinium enhancement (44% vs. 27%, p = 0.005). Men had significantly lower levels of FABP4 (18.36 ng/ml vs. 30.69 ng/ml, p = 0.001) and higher hs-cTnT levels (10 ng/L vs. 8 ng/L, p = 0.007).

**Table 1:** Baseline characteristics

	All (n = 331)	Male (n = 236)	Female (n = 95)	p-value
Baseline characteristics				
Age, mean (sd)	63.1 (12.6)	62.4 (12.9)	64.6 (11.8)	0.156
Hypertension, n (%)	246 (74.8)	176 (74.9)	70 (74.5)	1.00
Diabetes, n (%)	57 (17.4)	47 (20.1)	10 (10.6)	0.06
Active smoking, n (%)	59 (18.0)	42 (17.9)	17 (18.3)	*< 0.001*
Coronary artery disease, n (%)	140 (42.6)	114 (48.5)	26 (27.7)	*0.002*
Late gadolinium enhancement, n (%)	129 (39.1)	104 (44.1)	25 (26.6)	*0.005*
Perfusion deficit, n (%)	65 (19.8)	49 (20.9)	16 (17.0)	0.525
Atrial fibrillation, n (%)	66 (20.2)	48 (20.5)	18 (19.6)	0.969
Body mass index (kg/m^2^), mean (sd)	27.5 (4.7)	27.7 (4.3)	27.0 (5.5)	0.21
Height (cm), mean (sd)	174.4 (9.7)	178.4 (7.7)	164.4 (6.3)	*< 0.001*
Weight (kg), mean (sd)	83.7 (16.6)	88.1 (15.2)	72.7 (14.6)	*< 0.001*
Heart rate beats per min, mean (sd)	68 [12]	68 [12]	68 [12]	0.947
Cardiac magnet resonance imaging variables				
LVMI (g/m^2^), mean (sd)	44.5 (14.9)	47.6 (15.0)	36.6 (11.1)	*< 0.001*
LVEF (%), mean (sd)	65.2 (6.5)	64.8 (6.7)	66.2 (5.9)	0.087
LVEDD (mm), mean (sd)	49.6 (6.5)	51.0 (6.5)	46.3 (5.3)	*< 0.001*
RVEDD (mm), mean (sd)	33.8 (6.8)	35.0 (6.5)	30.9 (6.9)	*< 0.001*
RWT, mean (sd)	0.30 (0.12)	0.31 (0.13)	0.28 (0.09)	*0.014*
Laboratory results				
NT-proBNP (ng/L), median [IQR]	179.6 [76.9, 389.6]	179.3 [71.9, 416.5]	181.5 [81.1, 328.3]	0.592
CRP (mg/dl), median [IQR]	0.2 [0.1, 0.4]	0.2 [0.1, 0.4]	0.2 [0.1, 0.4]	0.568
hs-cTnT (ng/L), median [IQR]	9 [5, 16]	10 [6, 17]	8 [5, 14]	*0.007*
Creatinin (µmol/L), mean (sd)	88.4 (61.9)	97.2 (70.7)	97.2 (26.5)	*< 0.001*
eGFR (ml/min), mean (sd)	91.6 (32.7)	91.5 (32.0)	92.0 (34.5)	0.884
FABP4 (ng/ml), median [IQR]	22.79 [13.84, 36.46]	18.36 [12.24, 31.33]	30.69 [20.96, 49.74]	*0.001*

Baseline characteristic of 331 patients undergoing cardiac magnetic resonance imaging stratified by sex. Data presented as percentage, mean, or median

Italic values indicate statistically significant differences between men and women (using the two-sided t test or Wilcoxon rank-sum test as appropriate), p-value < 0.05

CRP, C- reactive protein; eGFR, estimated glomerular filtration rate; FABP4, Fatty-acid binding protein 4; hs-cTnT, cardiac high sensitivity troponin T; LVEDD, left ventricular end-diastolic diameter; LVEF, left ventricular ejection fraction; LVMI, left ventricular mass index; NT-proBNP, N-terminal pro B-type brain natriuretic peptid; RVEDD, right ventricular end-diastolic diameter; RWT, relative wall thickness

### Baseline characteristic stratified by FABP4 median

Patients with FABP4 levels above the median (22.79 ng/ml) were more likely female (41% vs. 16%, p < 0.001) and were older (66 years vs. 60 years, p < 0.001), presented with a higher rate of hypertension (85% vs. 64%, p < 0.001) and diabetes (23% vs. 12%, p = 0.018), a higher BMI (29 kg/m^2^ vs. 26 kg/m^2^, p < 0.001), a lower eGFR (79 ml/min vs. 105 ml/min, p < 0.001), higher hs-cTnT levels (12 ng/L vs. 8 ng/L p < 0.001), higher NT-proBNP levels (180 ng/L vs. 113 ng/L, p < 0.001), and higher CRP levels (0.2 mg/dl vs. 0.1 mg/dl, p < 0.001). Patients with higher FABP4 levels had significantly lower LVMI (41 g/m^2^ vs 48 g/m^2^, p < 0.001) and lower LVEDD levels (49 mm vs 51 mm, p = 0.002), but higher RWT (0.31 vs 0.28, p = 0.008). There was no difference regarding the presence of late gadolinium enhancement, perfusion deficit, or atrial fibrillation or regarding LVEF or RVEDD between both groups (Additional file 1: Table S1).

Due to the difference in FABP4 levels regarding men and women, we performed additional analyses stratified by the respective sex-specific median separately in men (median 13.81 ng/L) and women (median 36.52 ng/L, Additional file 1: Table S2). Similar to the overall cohort, in the smaller group of women, women with FABP4 levels above the median presented with a higher rate of hypertension (86% vs. 63%, p = 0.028), a higher BMI (30 kg/m^2^ vs. 24 kg/m^2^, p < 0.001), higher hs-cTnT levels (11 ng/L vs. 6 ng/L, p = 0.002) and a lower eGFR (85 ml/min vs. 100 ml/min, p = 0.04), but we could not observe differences in NT-proBNP or CRP levels, and there were no differences between the number of patients with late gadolinium enhancement, perfusion deficit, or atrial fibrillation. With respect to the CMR measures, women with FABP4 levels above the median had higher RVEDD (32 mm vs. 29 mm, p = 0.031) levels, but we did observe any other differences (Additional file 1: Table S2A).

We observed similar differences as seen in the overall cohort for men with FABP4 levels below and above the sex-specific median. Patients with FABP4 levels above the sex-specific median were older (66 years vs. 59 years, p < 0.001), had a higher rate of hypertension (85% vs. 65%, p = 0.001), late gadolinium enhancement (52% vs. 36%, p = 0.026), and diabetes (30% vs. 10%, p < 0.001) and were less likely smokers (16% vs. 20%, p = 0.008), had a higher BMI (29 kg/m^2^ vs. 26 kg/m^2^, p < 0.001), lower LVMI (45 g/m^2^ vs. 50 g/m^2^, p = 0.029), higher RWT (0.33 vs. 0.29, p = 0.008), higher NT-proBNP levels (240 ng/L vs 111 ng/L, p < 0.001), higher CRP levels (0.2 vs. 0.1, p < 0.001), higher hs-cTnT levels (14 ng/l vs. 8 ng/L, p < 0.001), and lower eGFR (80 ml/min vs 104 ml/min, p < 0.001, Additional file 1: Table S2B).

### Correlation analysis

In the Spearman-rank correlation analyses (in the overall cohort) FABP4 levels were significantly, negatively correlated with LVMI (r = − 0.25, p < 0.001), LVEDD (r = − 0.2, p < 0.001) and eGFR (r = − 0.48, p < 0.001) and positively with RWT (r = 0.2, p = 0.006), hs-cTnT (r = 0.36, p < 0.001) and NT-proBNP (r = 0.35, p < 0.001), age (r = 0.29, p < 0.001), and BMI (r = 0.36, p < 0.001, Table 2a and b). Overall, we observed stronger correlations between FABP4 levels and cardiac biomarkers compared to CMR measures.

**Table 2:** Spearman-rank correlation between FABP4 and CMR measures (A) and laboratory results, age, and body mass index (B) overall, in men, and in women

A	LVMI	RWT	LVEDD	LVEF	RVEDD
FABP 4 overall					
R	*− 0.25*	*0.15*	*− 0.20*	0.09	0.03
p-value	*< 0.001*	*0.006*	*< 0.001*	0.104	0.568
FABP 4 women					
R	**− **0.16	0.18	**− **0.05	0.12	*0.22*
p-value	0.136	0.076	0.661	0.243	*0.032*
FABP 4 men					
R	*− 0.17*	*0.21*	**− **0.12	0.04	0.08
p-value	*0.008*	*< 0.001*	0.068	0.588	0.211

B	Nt-proBNP	Hs-cTnT	eGFR	age	BMI
FABP 4 overall					
R	0.35	0.35	0.36	− 0.48	0.29
p-value	< 0.001	< 0.001	< 0.001	< 0.001	< 0.001
FABP 4 women					
R	0.18	0.18	0.46	−0.39	0.28
p-value	0.093	0.093	< 0.001	< 0.001	0.007
FABP 4 men					
R	0.40	0.40	0.42	− 0.51	0.30
p-value	< 0.001	< 0.001	< 0.001	< 0.001	< 0.001

Spearman Rank correlation analyses between FABP4 and CMR measures (A) and laboratory results, age, and body mass index (B) overall, in men, and in women

Italic values indicate statistically significant correlations, p-value < 0.05

BMI, body mass index; eGFR, estimated glomerular filtration rate; FABP4, Fatty-acid binding protein 4; hs-cTnT, cardiac high sensitivity troponin T; LVEDD, left ventricular end-diastolic diameter; LVEF, left ventricular ejection fraction; LVMI, left ventricular mass index; NT-proBNP, N-terminal pro B-type brain natriuretic peptid; RVEDD, right ventricular end-diastolic diameter; RWT, relative wall thickness

In women, FABP4 levels were significantly, positively correlated with RVEDD (r = 0.22, p = 0.032), with hs-cTnT (r = 0.46, p < 0.001), BMI (r = 0.41, p < 0.001), age (r = 0.29, p = 0.007) and negatively with eGFR (r = 0.41, p < 0.001), but there were no correlation between FABP4 and LVMI, RWT or NT-proBNP (Table 2a, b).

In men, higher FABP4 levels were significantly, negatively correlated with LVMI (r = − 0.17, p = 0.008) and eGFR (r = − 0.51, p < 0.001) and positively with RWT (r = 0.21, p < 0.001), hs-cTnT (r = 0.42, p < 0.001) and NT-proBNP (r = 0.40, p < 0.001), age (r = 0.30, p < 0.001), and BMI (r = 0.46, p < 0.001, Table 2a, b).

When the patients were stratified by FABP4 levels above and below the median (Additional file 1: Table S3 A and B) we observed a significant correlation between FABP4 and LVMI in patients with FABP4 levels below the median only (r = − 0.17, p = 0.029). In contrast, only in patients with FABP4 levels above the median FABP4 was significantly correlated with NT-proBNP (r = 0.29, p < 0.001), hs-cTnT (r = 0.38, p < 0.001) and eGFR (r = − 0.40, p < 0.001).

### Association between FABP4 and CMR-indices

In multivariable analyses, FABP4 levels were inversely associated with LVMI (est. − 2.93, p < 0.001) and LVEDD (est. − 1.15, p = 0.002) but positively with RWT (est. 0.02, p = 0.007). Only the associations between FABP4 and LVMI remained statistically significant in a fully adjusted model (Table 3). We did not observe any associations between FABP4 and RVEDD or LVEF.

**Table 3:** Associations between FABP4 and cardiac magnet resonance imaging variables

	Estimate	p-value
Unadjusted		
LVMI	− *2.93*	*< 0.001*
LVEF	0.51	0.153
LVEDD	*− 1.15*	*0.002*
RWT	*0.02*	0.007*
RVEDD	0.37	0.329
Fully adjusted		
LVMI	− *3.35*	*0.002*
RWT	0.0045	0.62
LVEDD	− 0.95	0.029

Estimates are beta coefficients per sex-standardized standard deviation increase of fatty acid binding protein 4

Models adjusted for age, sex, body mass index, heart rate, glomerular filtration rate, N-terminal pro B-type brain natriuretic peptid levels, and diagnosis of diabetes mellitus, arterial hypertension, or coronary artery disease

Italic values indicate statistically significant associations, Bonferroni corrected p-value ≤ 0.01

LVEDD, left ventricular end-diastolic diameter; LVEF, left ventricular ejection fraction; LVMI, left ventricular mass index; RVEDD, right ventricular end-diastolic diameter; RWT, relative wall thickness

In women, we did not observe any significant associations between FABP4 and any of the CMR -Indices (Additional file 1: Table S4). We did not observe a significant association between FABP4 and LVMI (est. − 1.5, p = 0.273) in a small subsample of overweight and obese women (BMI ≥ 25, n = 58).

**Fig. 2:**
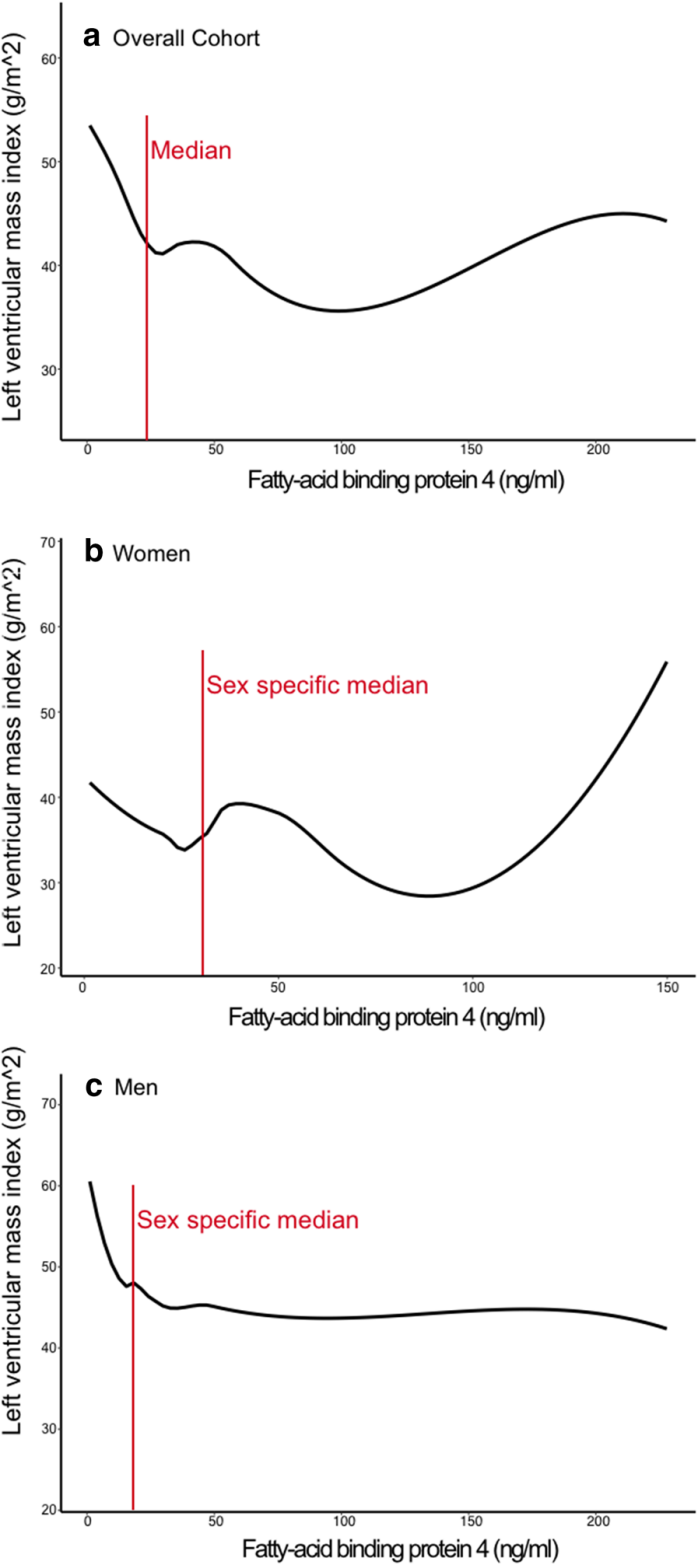
Relationship between FABP4 and LVMI. Relationship between FABP4 (fatty-acid binding protein 4) levels and LVMI (left ventricular mass index) overall (**a**), in women (**b**), and in men (**c**)

In contrast, in men we observed a significant association between FABP4 and LVMI (est. − 2.5, p = 0.01) and RWT (est.0.03, p = 0.003) in the unadjusted model and FABP4 was still associated with LVMI in in the fully adjusted model (est. − 4.84, p < 0.001, Additional file 1: Table S4). The relationship between FABP4 and LVMI in the overall cohort, in women, and in men is depicted in Fig. 2a–c.

When the study sample was stratified in patients with FABP4 levels above and below the median, we did not observe any associations between FABP4 and the CMR indices except of an inverse association between FABP and LVEDD in patients with lower FABP4 levels, which could not be observed in the fully adjusted model (Additional file 1: Table S5).

### Secondary analyses—associations between FABP4 and NT-proBNP and hs-cTnT

FABP4 levels were not significantly associated with hs-cTnT in secondary analyses (est. 9.5, p = 0.289) but we observed a strong, positive association between FABP4 and NT-proBNP levels (est. 248.7, p < 0.001). This association remained statistically significant in the fully adjusted model (est. 150.7, p = 0.005, Additional file 1: Table S6).

**Fig. 3:**
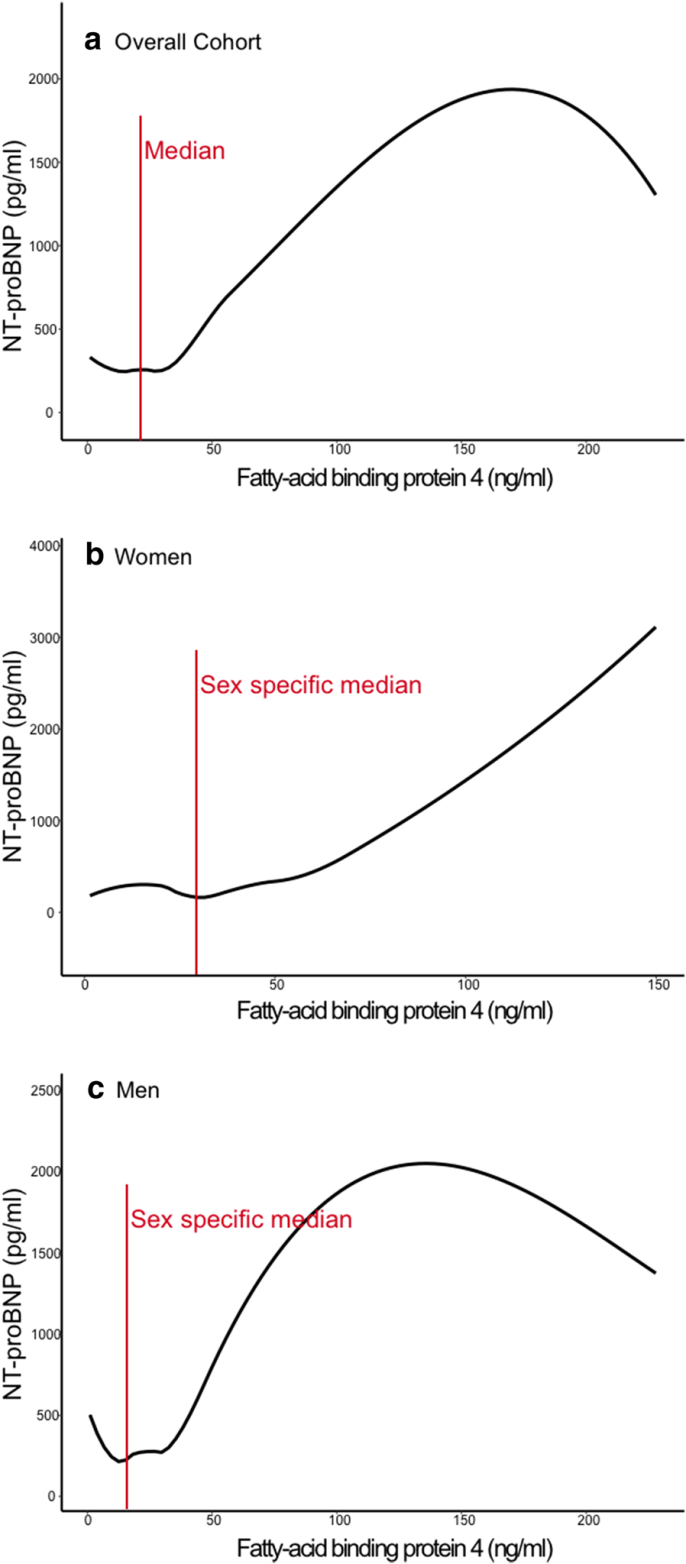
Relationship between FABP4 and NT-proBNP. Relationship between FABP4 (fatty-acid binding protein 4) levels and NT-proBNP (N-terminal pro B-type brain natriuretic peptid) levels overall (**a**), in women (**b**), and in men (**c**)

When the study sample was stratified by FABP4 levels below and above the median, we did not observe an association between FABP4 and NT-pro BNP or hs-cTnT in patients with low FABP4 levels (Additional file 1: Table S7) but a significant association between FABP4 and NT-pro BNP in patients with high FABP4 levels (es. 720.3, p < 0.001) which was still significant in the fully adjusted model (est. 680.6, p < 0.001). The relationship between FABP4 and NT-proBNP in the overall cohort, in women, and in men is depicted in Fig. 3a–c.

When the study sample was divided between men and women, we observed a significant association between FABP4 and NT-pro BNP both in men (est. 242.9, p < 0.001, Additional file 1: Table S8) and in women (est. 298.7, p < 0.001, Additional file 1: Table S8). However, this association only remain significant in the fully adjusted model in women.

## Discussion

### Principal findings

In the present study, we investigated the associations between the adipokine FABP4 and measures of cardiac remodeling and function using state-of-the art CMR images and, in secondary analyses, between FABP4 and cardiac biomarkers. Our principal findings are four-fold:

First, we observed that patients with higher FABP4 levels (above the median) had lower values of LVMI and LVEDD (possible beneficial relationship) but higher values of RWT and higher levels of hs-cTnT and NT-proBNP (possible adverse relationship).

Second, FABP4 levels correlated negatively with LVMI and LVEDD (again, a possible beneficial correlation), but positively with hs-cTnT and NT-proBNP, which might show an adverse correlation. The correlation between FABP4 and LVMI was only observed in patients with FABP4 levels below the median, while the correlation between FABP4 and NT-proBNP levels was only observed in patients with FABP4 levels above the median. This observation might show, that FABP4 has different, dose dependent, correlations with LVMI and NT-proBNP.

Third, in multivariable regression analyses, FABP4 was inversely associated with LVMI in the fully adjusted model (possible beneficial association) but FABP4 was positively associated with NT-proBNP, a possible adverse association. The association between FABP4 and NT-proBNP was only observed in patients with FABP4 levels above the median, which again might suggest a dose-dependent association. Unfortunately, we did not observe significant associations between FABP4 and LVMI when the study sample was stratified in patients with FABP4 levels below and above the median.

Forth, there were different correlations and associations between sexes. When the study sample was stratified by the respective sex-specific median, only men showed similar differences between the two groups regarding LVMI and NT-proBNP as the overall cohort, while there were no differences in women. FABP4 levels only correlated positively with NT-proBNP and negatively with LVMI in men, but we did not observe similar correlations in women. Only in men we observed a significant association between FABP4 and LVMI in multivariable regression, while there was no significant association in women. Interestingly, we observed a significant association between FABP4 and NT-proBNP in both sexes in the unadjusted model, which only remained significant in the fully adjusted model in women.

### Comparisons with the published literature

Our observation, that FABP4 was inversely associated with LVMI is in line with and has only been reported by one other study. Similar to our findings, a study from the Framingham Heart Study reported such a, potential beneficial, association in a large population-based cohort [21]. However, several other publications have reported potential harmful associations. Baessler et al. have reported, that FABP4 was positively associated with LVMI and furthermore with diastolic dysfunction. As the study was carried out in morbidly obese with metabolic syndrome, the results on LVMI are only partly comparable with our study, as our patients had a mean BMI of 27 kg/m^2^ and only 17% were diabetic. [16] Unfortunately we cannot make any statements regarding diastolic function, as we did not use echocardiographic variables. Therefore, we also cannot compare our results to another publication, which has reported an association between FABP4 and diastolic dysfunction [17]. The same study did not find a significant correlation between FABP4 and LVMI, but was carried out in a population-based study with fewer comorbidities compared to our study. Two studies reported potential adverse associations between FABP4 and markers of left ventricular hypertrophy and function, whereas we observed a potential beneficial association between FABP4 and LVMI and no association between FABP4 and LVEF. However, as one study was carried out in patients with sleep apnea [18] and the other one in patients with coronary artery disease, [20] the results cannot be compared directly. Interestingly, another publication investigated the association between FABP4 and LVMI in obese and overweight women also using CMR and reported a potential harmful association between FABP4 and LVMI. Therefore, we carried out an analysis in obese and overweight women only, where we did not find a statistically significant association. As the mean age in the respective study was about 20 years younger compared to our study sample, again, the study is only partly comparable with our results [19].

While our results on LVMI might reflect a potential beneficial association with cardiac remodeling, our results on NT-proBNP reflect a potential harmful association. This is, in contrast to our observations on LVMI, in line with other publications: Cabre et al. reported, that FABP4 levels were higher in patients with heart failure and correlated with NT-proBNP levels and heart failure severity. However, the mean LVEF was 32% compared to 65% in our study. [14] Another study reported, that FABP4 levels were higher in patients with compared to patients without heart failure and FABP4 levels correlated with NT-proBNP, [13] and FABP4 has been described to be associated with incidence of heart failure [15]. However, as our study was cross-sectional, we cannot comment on heart failure incidence. A recent publication reported an association between FABP4 and atrial fibrillation [9], however in our study sample the incidence of atrial fibrillation was only about 20% and we did not observe different rates of atrial fibrillation in patients with lower or higher FABP4 levels.

The observation, that FABP4 levels are higher in women compared to men has been reported by several other studies [15, 20, 21, 23]. To the best of our knowledge, we are the first to report possible differences between the association of FABP4 with cardiac remodeling in men and women. In most of our analyses it seems that the association between FABP4 and LVMI and NT-proBNP is only present in men, as only men showed a significant correlation between FABP4 and LVMI and NT-proBNP and only men showed a significant association between FABP4 and LVMI in multivariable analysis. But as only women showed a significant association between FABP4 and NT-proBNP in the fully adjusted model in the multivariable analyses, there seems to be an association in women also, so no conclusion can be drawn based on our results. Different associations between men and women for FABP4 have already been described for coronary artery disease and carotid atherosclerosis: FABP4 was more closely related with coronary artery disease in women compared to men [24] and FABP4 had a greater impact on coronary atherosclerosis in women [25]. Furthermore, FABP4 was a determinant of carotid atherosclerosis in women but not men [26]. However, in a different publication, FABP4 was described to be independently associated with coronary artery disease in men (but no women have been included in the investigation). Therefore, no final conclusions on sex-differences regarding possible FABP4 associations can be drawn, but further studies are needed to gain insights in possible sex-differences.

### Possible dose-dependent associations

The observation, that higher FABP4 levels are associated with lower LVMI but higher NT-proBNP levels is counterintuitive. The first association would indicate a potentially beneficial effect of FABP4 on the heart whereas the latter association would indicate a potentially harmful association. In Fig. 2 one can appreciate that the inverse, potentially beneficial, relation between FABP4 and LVMI is most pronounced in FABP4 levels below the (sex-specific) median and that there might even been a rise in LVMI with very high FABP4 levels, especially in women. The potentially harmful relation between FABP4 and NT-proBNP seems to begin in FABP4 levels above the (sex-specific) median (Fig. 3). Furthermore, we observed the potential beneficial correlation between FABP4 and LVMI only in patients with lower FABP4 levels but the potential harmful correlation between FABP4 and NT-proBNP only in patients with higher FABP4 levels. And the association between FABP4 and NT-proBNP was only observed in patients with FABP4 levels above the median. Taken all these findings together, even if we did not observe significant associations between FABP4 levels and LVMI when the study sample was stratified in patients with FABP4 levels below and above the median, our observations point out to a possible dose-dependency of FABP4. It seems, that lower FABP4 levels might be beneficial while higher levels might be harmful. This observation might be explained by end-organ resistance; FABP4 might have a cardioprotective effect on the heart, but with increased FABP4 levels (e.g. due to obesity), this effect diminishes. Another explanation for this finding could be, that FABP4 differently affects the heart based on underlying conditions. Here, Zhang et al. investigated the effects of FABP4 overexpression on the myocardium in mice in relation to aorta constriction and observed that only in the presence of aorta constriction, FABP4 was associated with myocardial hypertrophy [27]. Baessler et al. reported an association between FABP4 and left ventricular diastolic dysfunction in obese individuals with metabolic syndrome but not in lean individuals or in obese individuals without metabolic syndrome [16].

Of course, the concept of a possible dose-dependent association between FABP4 and the myocardium is completely speculative, and the majority of the publications so far does not support a beneficial association of effect of FABP4 not only on the heart but other systems: FABP4 has been described to have adverse association with insulin resistance, diabetes and the metabolic syndrome, [3–6], and with atherosclerosis and cardiovascular diseases, [1, 7] and sodium glucose cotransporter 2 inhibitors might have a beneficial effect via decreased FABP4 expression on perivascular adipose tissue [10]. FABP4 from epicardial fat might have adverse effects on coronary artery disease and atrial fibrillation. [8, 9] Also basic research studies have shown that FABP4 suppresses contraction of cardiomyocytes [11] and increase intracellular lipid accumulation in cardiomyocytes [12]. However, even in light of this overwhelming evidence of potential harmful associations, a potential beneficial, dose-dependent association on the myocardium needs further investigation, especially as FABP4-inhibition or FABP4-receptor-blocking have been discussed as possible therapeutic target in the metabolic syndrome [22].

## Limitations

Our study has several limitations. The sample size was rather small, especially when we divided the study sample by sex and by FABP4 levels below and above the median. Hence, our findings need to be replicated in larger cohorts to draw robust conclusions. Here it would be very interesting to investigate the patterns of low and high FABP4 levels in men and women separately. Due to our small cohort, we were not able to perform such analyses. Our observation that FABP4 was associated with NT-proBNP levels was derived from secondary analyses only and therefore needs further validation, even when similar results have already been published, as the previous cohorts were not entirely comparable to our cohort. Furthermore, we have to assume that almost all of the analyzed women were postmenopausal, therefore we cannot conclude any observations on premenopausal women. As our study design is cross-sectional, it is not suitable to analyze cause-effect relationships.

## Conclusions

We could observe a potential beneficial association between FABP4 and LVMI (most pronounced with lower FABP4 concentrations) but a potential harmful association between FABP4 and NT-proBNP levels (most pronounced with higher FABP4 concentrations). The underlying mechanism remain unclear and the effects of FABP4 on the myocardium a matter of further investigation, which is of high importance, as FABP4-inhibition is discussed as a potential therapeutic target.

## Supplementary information

**Additional file 1: Table S1.** Baseline characteristics stratified by FABP4 median. **Table S2.** Sex-specific Baseline characteristics stratified by FABP4. **Table S3.** A and B Spearman-rank correlation between FABP4 and CMR measures (A) and laboratory results, age, and body mass index (B) overall, in patients with FABP4 levels below and above the median. **Table S4.** Associations between FABP4 and cardiac magnet resonance imaging variables in women and men. **Table S5.** Associations between FABP4 and cardiac magnet resonance imaging variables in patients with FABP4 levels above or below the median. **Table S6.** Associations between FABP4 and NT-pro BNP and hs-cTnT in the overall cohort. **Table S7.** Associations between FABP4 and NT-pro BNP and hs-cTnT in patients with FABP4 levels below or above the median. **Table S8.** Associations between FABP4 and NT-pro BNP and hs-cTnT in man and women.

## Data Availability

The data that support the findings of this study are not available due to containing information that could compromise research participant privacy/consent.

## Abbreviations

BMI: Body mass index
CMR: Cardiac magnetic resonance imaging (CMR)
CRP: C-reactive Protein
ELISA: Enzyme-linked immunosorbent assay
FABP4: Fatty-acid binding protein 4
eGFR: Estimated glomerular filtration rate
hs-cTnT: Cardiac high sensitivity troponin T
LVEDD: Left ventricular end-diastolic diameter
LVEF: Left ventricular ejection fraction
LVMI: Left ventricular mass index
NT-proBNP: N-terminal pro B-type natriuretic peptide
RVEDD: Right ventricular end-diastolic diameter
RWT: Relative wall thickness
