# Supplementary material for: The adipokine fatty-acid binding protein 4 and cardiac remodeling
Source: Cardiovasc Diabetol. 2020 Jul 29;19:117. doi: 10.1186/s12933-020-01080-x (PMC7392717; doi:10.1186/s12933-020-01080-x)
Supplement: Supplementary file 1 — Additional file 1: Table S1. Baseline characteristics stratified by FABP4 median. Table S2. Sex-specific Baseline characteristics stratified by FABP4. Table S3. A and B Spearman-rank correlation between FABP4 and CMR measures (A) and laboratory results, age, and body mass index (B) overall, in patients with FABP4 levels below and above the median. Table S4. Associations between FABP4 and cardiac magnet resonance imaging variables in women and men. Table S5. Associations between FABP4 and cardiac magnet resonance imaging variables in patients with FABP4 levels above or below the median. Table S6. Associations between FABP4 and NT-pro BNP and hs-cTnT in the overall cohort. Table S7. Associations between FABP4 and NT-pro BNP and hs-cTnT in patients with FABP4 levels below or above the median. Table S8. Associations between FABP4 and NT-pro BNP and hs-cTnT in man and women. [file 12933_2020_1080_MOESM1_ESM.docx]

**Additional file 1**

**Additional Tables**

**Table S1.** Baseline characteristics stratified by FABP4 median

|  | **All**  (n=331) | **FABP4 ≤ Median**  (n=166) | **FABP4 > Median**  (n=165) | p-value |
| --- | --- | --- | --- | --- |
| **Baseline characteristics** | | | | |
| Female Sex, n (%) | 95 (28.7) | 27 (16.4) | 67 (40.6) | **<0.001*** |
| Age, mean (sd) | 63.1 (12.6) | 60.0 (12.5) | 66.0 (12.0) | **<0.001*** |
| Hypertension, n (%) | 246 (74.8) | 105 (64.4) | 140 (84.8) | **<0.001*** |
| Diabetes, n (%) | 57 (17.4) | 20 (12.2) | 37 (22.7) | **0.018*** |
| Smoking, n (%) | 59 (18.0) | 35 (21.6) | 24 (14.6) | 0.23 |
| Coronary artery disease, n (%) | 140 (42.6) | 68 (41.7) | 71 (43.0) | 0.59 |
| Late Gadolinium Enhancement, n (%) | 129 (39.1) | 60 (36.4) | 69 (41.8) | 0.367 |
| Perfusion Deficit, n (%) | 65 (19.8) | 36 (21.8) | 29 (17.7) | 0.422 |
| Atrial fibrillation, n (%) | 66 (20.2) | 27 (16.7) | 39 (23.8) | 0.144 |
| Body mass index (kg/m^2^), mean (sd) | 27.5 (4.7) | 26.0 (3.7) | 28.9 (5.1) | **<0.001*** |
| Height (cm), mean (sd) | 174.4 (9.7) | 176.7 (9.1) | 172.2 (9.6) | **<0.001*** |
| Weight (kg), mean (sd) | 83.7 (16.6) | 81.5 (14.6) | 85.9 (18.2) | **0.017*** |
| Heart rate, mean (sd) | 68 12) | 67 (12) | 69 (12) | 0.24 |
| **Cardiac magnet resonance imaging variables** | | | | |
| LVMI (g/m^2^), mean (sd) | 44.5 (14.9) | 47.5 (14.6) | 41.4 (14.6) | **<0.001*** |
| LVEF (%), mean (sd | 65.2 (6.5) | 64.6 (6.4) | 65.8 (6.6) | 0.101 |
| LVEDD (mm), mean (sd) | 49.6 (6.5) | 50.8 (5.9) | 48.6 (6.9) | **0.002*** |
| RVEDD (mm), mean (sd) | 33.8 (6.8) | 33.7 (6.7) | 34.0 (7.0) | 0.70 |
| RWT, mean (sd) | 0.30 (0.12) | 0.28 (0.10) | 0.32 (0.14) | **0.008*** |
| **Laboratory results** | | | | |
| NT-proBNP (ng/L), median [IQR] | 179.6 [76.9, 389.6] | 112.8 [51.9, 230.6] | 237.7 [119.1, 465.5] | **<0.001*** |
| CRP (mg/dl), median [IQR] | 0.2 [0.1, 0.4] | 0.1 [0.1, 0.3] | 0.2 [0.1, 0.5] | **<0.001*** |
| Hs-cTnT (ng/L), median [IQR] | 9 [5, 16] | 8 [5, 11] | 12 [7, 21] | **<0.001*** |
| Creatinin (µmol/L), mean (sd) | 88.4 (61.9) | 70.7 (17.7) | 97.2 (79.6) | **<0.001*** |
| eGFR (ml/min), mean (sd) | 91.6 (32.7) | 105.2 (26.4) | 78.9 (32.9) | **<0.001*** |

Baseline characteristic of 331 patients undergoing cardiac magnetic resonance imaging stratified by FABP4 median (22.79ng/ml). Data presented as percentage, mean, or median. * indicates statistically significant differences between the two groups (using the two-sided t test or Wilcoxon rank-sum test as appropriate).

Abbreviations: CRP: C- reactive protein; eGFR: estimated glomerular filtration rate; FABP4: Fatty-acid binding protein 4; hs-cTnT: cardiac high sensitivity troponin T; LVEDD: left ventricular end-diastolic diameter; LVEF: left ventricular ejection fraction; LVMI: left ventricular mass index; NT-proBNP: N-terminal pro B-type brain natriuretic peptid; RVEDD: right ventricular end-diastolic diameter; RWT: relative wall thickness.

**Table S2.** Sex-specific Baseline characteristics stratified by FABP4

| **A** | **Women**  (n=95) | **FABP4 ≤ Median**  (n=48) | **FABP4 > Median**  (n=47) | p-value |
| --- | --- | --- | --- | --- |
| **Baseline characteristics** | | | | |
| Age, mean (sd) | 64.6 (11.8) | 63.1 (11.2) | 65.7 (12.2) | 0.28 |
| Hypertension, n (%) | 70 (74.5) | 29 (63.0) | 40 (85.1) | **0.028*** |
| Diabetes, n (%) | 10 (10.6) | 3 (6.4) | 7 (15.2) | 0.30 |
| Smoking, n (%) | 17 (18.3) | 12 (26.1) | 5 (10.9) | 0.12 |
| Coronary artery disease, n (%) | 26 (27.7) | 11 (23.9) | 14 (29.8) | 0.9 |
| Late Gadolinium Enhancement, n (%) | 25 (26.6) | 9 (19.1) | 16 (34.0) | 0.161 |
| Perfusion Deficit, n (%) | 16 (17.0) | 6 (12.8) | 10 (21.3) | 0.410 |
| Atrial fibrillation, n (%) | 18 (19.6) | 8 (17.4) | 10 (21.7) | 0.793 |
| Body mass index (kg/m^2^), mean (sd) | 27.0 (5.5) | 24.2 (4.1) | 29.5 (5.4) | **<0.001*** |
| Height (cm), mean (sd) | 164.4 (6.3) | 164.3 (6.3) | 164.8 (6.2) | 0.71 |
| Weight (kg), mean (sd) | 72.7 (14.6) | 65.1 (10.4) | 80.1 (14.5) | **<0.001*** |
| Heart rate, mean (sd) | 68 (12) | 70 (13) | 66 (11) | 0.17 |
| **Cardiac magnet resonance imaging variables** | | | | |
| LVMI (g/m^2^), mean (sd) | 36.7 (35.8) | 35.8 (8.8) | 37.2 (13.2) | 0.545 |
| LVEF (%), mean (sd | 66.2 (5.9) | 66.0 (6.1) | 66.4 (5.8) | 0.74 |
| LVEDD (mm), mean (sd) | 46.3 (5.3) | 45.5 (4. 7) | 47.1 (5.8) | 0.16 |
| RVEDD (mm), mean (sd) | 30.9 (6.9) | 29.4 (6.0) | 32.4 (7. 4) | **0.031*** |
| RWT, mean (sd) | 0.28 (0.09) | 0.27 (0.08) | 0.28 (0.09) | 0.427 |
| **Laboratory results** | | | | |
| NT-proBNP (ng/L), median [IQR] | 181.5 [81.1, 328.3] | 188.3 [80.1, 307.0] | 176.5 [95.4, 322.2] | 0.79 |
| CRP (mg/dl), median [IQR] | 0.2 [0.1, 0.4] | 0.1 [0.1, 0.4] | 0.2 [0.1, 0.4] | 0.16 |
| Hs/cTnT (ng/L), median [IQR] | 8 [5, 14] | 6 [4, 8] | 11 [5., 18] | **0.002*** |
| Creatinin (µmol/L), mean (sd) | 68.1 (26.5) | 60.1 (8.8) | 74.3 (26.5) | **0.002*** |
| eGFR (ml/min), mean (sd) | 92.0 (34.5) | 99.8 (30.4) | 85.4 (36.6) | **0.040*** |

| **B** | **Men**  (n=236) | **FABP4 ≤ Median**  (n=118) | **FABP4 > Median**  (n=118) | p-value |
| --- | --- | --- | --- | --- |
| **Baseline characteristics** | | | | |
| Age, mean (sd) | 62.4 (12.9) | 58.9 (13.0) | 66.0 (11.9) | **<0.001*** |
| Hypertension, n (%) | 176 (74.9) | 76 (65.0) | 100 (84.7) | **0.001*** |
| Diabetes, n (%) | 47 (20.1) | 12 (10.3) | 35 (29.9) | **<0.001*** |
| Smoking, n (%) | 42 (17.9) | 23 (19.8) | 19 (16.1) | **0.008*** |
| Coronary artery disease, n (%) | 114 (48.5) | 53 (45.3) | 61 (51.7) | 0.34 |
| Late Gadolinium Enhancement, n (%) | 104 (44.1) | 43 (36.4) | 61 (51.7) | **0.026*** |
| Perfusion Deficit, n (%) | 49 (20.9) | 25 (21.2) | 24 (20.5) | 1.000 |
| Atrial fibrillation, n (%) | 48 (20.5) | 20 (17.2) | 28 (23.7) | 0.286 |
| Body mass index (kg/m^2^), mean (sd) | 27.7 (4.3) | 26.1 (3.3) | 29.3 (4.6) | **<0.001*** |
| Height (cm), mean (sd) | 178.4 (7.7) | 179.2 (8.0) | 177.5 (7.6) | 0.08 |
| Weight (kg), mean (sd) | 88.1 (15.2) | 84.1 (13.3) | 92.2 (16.0) | **<0.001*** |
| Heart rate, mean (sd) | 68 (11) | 68 (11) | 69 (13) | 0.54 |
| **Cardiac magnet resonance imaging variables** | | | | |
| LVMI (g/m^2^), mean (sd) | 47.6 (15.0) | 49.7 (15.3) | 45.5 (14.5) | **0.029*** |
| LVEF (%), mean (sd) | 64.8 (6.7) | 64.8 (6.3) | 64.8 (7.0) | 0.94 |
| LVEDD (mm), mean (sd) | 51.0 (6.5) | 51.6 (5.7) | 50.5 (7.2) | 0.191 |
| RVEDD (mm), mean (sd) | 35.0 (6.5) | 34.5 (6.8) | 35.5 (6.1) | 0.23 |
| RWT, mean (sd) | 0.31 (0.13) | 0.29 (0.09) | 0.33 (0.15) | **0.008*** |
| **Laboratory results** | | | | |
| NT-proBNP (ng/L), median [IQR] | 179.3 [71.9, 416.5] | 110.7 [47.5, 216.4] | 240.3 [110.7, 485.4] | **<0.001*** |
| CRP (mg/dl), median [IQR] | 0.2 [0.1, 0.4] | 0.1 [0.1, 0.3] | 0.2 [0.1, 0.5] | **<0.001*** |
| hs-cTnT (ng/L), median [IQR] | 10 [6, 17] | 8 [8, 11] | 14 [8, 27] | **<0.001*** |
| Creatinin (µmol/L), mean (sd) | 94.6 (66.3) | 75.1 (15.0) | 112.3 (88.4) | **<0.001*** |
| eGFR (ml/min), mean (sd) | 91.5 (32.0) | 103.8 (25.2) | 79.8 (33.5) | **<0.001*** |

Baseline characteristic of 95 female (**A**) and 236 male (**B**) patients undergoing cardiac magnetic resonance imaging stratified by sex specific FABP4 median (female: 36.52 ng/ml; male: 13.81ng/ml). Data presented as percentage, mean, or median. * indicates statistically significant differences between the two groups (using the two-sided t test or Wilcoxon rank-sum test as appropriate).

Abbreviations: CRP: C-reactive protein; eGFR: estimated glomerular filtration rate; FABP4: Fatty-acid binding protein 4; hs-cTnT: cardiac high sensitivity troponin T; LVEDD: left ventricular end-diastolic diameter; LVEF: left ventricular ejection fraction; LVMI: left ventricular mass index; NT-proBNP: N-terminal pro B-type brain natriuretic peptid; RVEDD: right ventricular end-diastolic diameter; RWT: relative wall thickness.

**Table S3 A and B.** Spearman-rank correlation between FABP4 and CMR measures (A) and laboratory results, age, and body mass index (B) overall, in patients with FABP4 levels below and above the median.

| A |  | | LVMI | RWT | LVEDD | LVEF | RVEDD |
| --- | --- | --- | --- | --- | --- | --- | --- |
| FABP 4 Overall | | R | **-0.25** | **0.15** | **-0.20** | 0.09 | 0.03 |
|  |  | p-value | **< 0.001** | **0.006** | **< 0.001** | 0.104 | 0.568 |
| FABP 4 ≤ median | | R | **-0.17** | -0.03 | -0.13 | -0.03 | -0.02 |
|  |  | p-value | **0.029** | 0.7127 | 0.074 | 0.731 | 0.843 |
| FABP 4 > median | | R | 0.001 | 0.15 | -0.01 | 0.06 | 0.11 |
|  |  | p-value | 0.9934 | 0.058 | 0.885 | 0.415 | 0.163 |

| B |  | Nt-proBNP | Hs-cTnT | eGFR | age | BMI |
| --- | --- | --- | --- | --- | --- | --- |
| FABP 4 Overall | R | **0.35** | **0.36** | **-0.48** | **0.29** | **0.36** |
|  | p-value | **< 0.001** | **< 0.001** | **< 0.001** | **< 0.001** | **< 0.001** |
| FABP 4 ≤ median | R | 0.15 | 0.13 | -0.09 | **0.23** | 0.022 |
|  | p-value | 0.075 | 0.1143 | 0.2641 | **0.003** | 0.778 |
| FABP 4 > median | R | **0.29** | **0.38** | **-0.40** | 0.12 | **0.32** |
|  | p-value | **< 0.001** | **< 0.001** | **< 0.001** | 0.1408 | **< 0.001** |

Spearman Rank correlation analyses between FABP4 and CMR measures (A) and laboratory results, age, and body mass index (B) overall, in patients with FABP4 levels below and above the median (22.79ng/ml).

Abbreviations: BMI: body mass index; eGFR: estimated glomerular filtration rate; FABP4: Fatty-acid binding protein 4; hs-cTnT: cardiac high sensitivity troponin T; LVEDD: left ventricular end-diastolic diameter; LVEF: left ventricular ejection fraction; LVMI: left ventricular mass index; NT-proBNP: N-terminal pro B-type brain natriuretic peptid; RVEDD: right ventricular end-diastolic diameter; RWT: relative wall thickness.

**Table S4.** Associations between FABP4 and cardiac magnet resonance imaging variables in women and men

| **Women** | **Estimate** | p-value |
| --- | --- | --- |
| LVMI | -0.27 | 0.83 |
| LVEF | 0.63 | 0.331 |
| LVEDD | -0.56 | 0.334 |
| RWT | 0.51 | 0.153 |
| RVEDD | 0.97 | 0.20 |

| **Men** | **Estimate** | p-value | |
| --- | --- | --- | --- |
| **LVMI** | **-2.53** | **0.01*** | |
| LVEF | 0.30 | 0.50 | |
| LVEDD | -0.77 | 0.07 | |
| **RWT** | **0.03** | **0.003*** | |
| RVEDD | 0.77 | 0.08 | |
| **Fully adjusted** | | |  |
| **LVMI** | **-4.84** | **<0.001** |  |
| RWT | 0.003 | 0.80 |  |

N= 95 women and n= 236 men. Estimates are beta coefficients per sex-standardized standard deviation increase of fatty acid binding protein 4. Models adjusted for age, body mass index, heart rate, glomerular filtration rate, N-terminal pro B-type brain natriuretic peptid levels, and diagnosis of diabetes mellitus, arterial hypertension, or coronary artery disease. * indicates p<0.05 in secondary analyses.

Abbreviations: LVEDD: left ventricular end-diastolic diameter; LVEF: left ventricular ejection fraction; LVMI: left ventricular mass index; RVEDD: right ventricular end-diastolic diameter; RWT: relative wall thickness.

**Table S5.** Associations between FABP4 and cardiac magnet resonance imaging variables in patients with FABP4 levels above or below the median

| **FABP4**≤**median** | **Estimate** | p-value | |
| --- | --- | --- | --- |
| LVMI | -3.1 | 0.064 | |
| LVEF | -0.29 | 0.693 | |
| **LVEDD** | **-1.51** | **0.0286** | |
| RWT | 0.03 | 0.769 | |
| RVEDD | 0.18 | 0.812 | |
| **Fully adjusted** | | |  |
| LVEDD | -0.64 | 0.361 |  |

| **FABP4>median** | **Estimate** | p-value |
| --- | --- | --- |
| LVMI | 0.59 | 0.743 |
| LVEF | 0.66 | 0.416 |
| LVEDD | 0.16 | 0.853 |
| RWT | 0.02 | 0.253* |
| RVEDD | 1.07 | 0.213 |

N = 166 patient with FABP4 levels ≤ median (22.79ng/ml) and n=165 patients with FABP4 levels below the median. Estimates are beta coefficients per sex-standardized standard deviation increase of fatty acid binding protein 4.

Abbreviations: FABP4: fatty acid binding protein 4; LVEDD: left ventricular end-diastolic diameter; LVEF: left ventricular ejection fraction; LVMI: left ventricular mass index; RVEDD: right ventricular end-diastolic diameter; RWT: relative wall thickness.

**Table S6**. Associations between FABP4 and NT-pro BNP and hs-cTnT in the overall cohort

|  | **Estimate** | p-value |
| --- | --- | --- |
| **Unadjusted** | | |
| **Nt-pro BNP** | **248.7** | **<0.001*** |
| Hs-cTnT | 9.5 | 0.289 |
| **Fully adjusted** | | |
| **Nt-pro BNP** | **150.7** | **0.005** |

Estimates are beta coefficients per sex-standardized standard deviation increase of fatty acid binding protein 4. * indicates p < 0.05. Models adjusted for age, sex, body mass index, heart rate, glomerular filtration rate, and diagnosis of diabetes mellitus, arterial hypertension, or coronary artery disease. NT-pro BNP and hs-cTnT have been modeled individually.

Abbreviations: FABP4: Fatty-acid binding protein 4; hs-cTnT: cardiac high sensitivity troponin T; NT-pro BNP: N-terminal pro B-type brain natriuretic peptid.

**Table S7**. Associations between FABP4 and NT-pro BNP and hs-cTnT in patients with FABP4 levels below or above the median

**FABP4**≤**median**

|  | **Estimate** | p-value |
| --- | --- | --- |
| **Unadjusted** | | |
| Nt-pro BNP | -24.1 | 0.635 |
| Hs-cTnT | 2.7 | 0.863 |

**FABP4**>**median**

|  | **Estimate** | p-value |
| --- | --- | --- |
| **Unadjusted** | | |
| **Nt-pro BNP** | **720.3** | **<0.001*** |
| Hs-cTnT | 37.2 | 0.110 |
| **Fully adjusted** | | |
| **Nt-pro BNP** | **680.6** | **<0.001*** |

N = 166 patient with FABP4 levels ≤ median (22.79ng/ml) and n=165 patients with FABP4 levels below the median. Estimates are beta coefficients per sex-standardized standard deviation increase of fatty acid binding protein 4. * indicates p < 0.05. NT-pro BNP and hs-cTnT have been modeled individually.

Abbreviations: FABP4: Fatty-acid binding protein 4; hs-cTnT: cardiac high sensitivity troponin T; NT-pro BNP: N-terminal pro B-type brain natriuretic peptid.

**Table S8**. Associations between FABP4 and NT-pro BNP and hs-cTnT in man and women.

**Men**

|  | **Estimate** | p-value |
| --- | --- | --- |
| **Unadjusted** | | |
| **Nt-pro BNP** | **242.9** | **<0.001** |
| Hs-cTnT | 0.012 | 0.999 |
| **Fully adjusted** | | |
| Nt-pro BNP | 64.6 | 0.286 |

**Women**

|  | **Estimate** | p-value |
| --- | --- | --- |
| **Unadjusted** | | |
| **Nt-pro BNP** | **298.7** | **<0.001*** |
| Hs-cTnT | 31 | 0.214 |
| **Fully adjusted** | | |
| Nt-pro BNP | **329.2** | **0.01*** |

N= 95 women and n= 236 men. Estimates are beta coefficients per sex-standardized standard deviation increase of fatty acid binding protein 4. * indicates p < 0.05. Models adjusted for age, body mass index, heart rate, glomerular filtration rate, and diagnosis of diabetes mellitus, arterial hypertension, or coronary artery disease. NT-pro BNP and hs-cTnT have been modeled individually.

Abbreviations: FABP4: Fatty-acid binding protein 4; hs-cTnT: cardiac high sensitivity troponin T; NT-pro BNP: N-terminal pro B-type brain natriuretic peptid.
